# The risk of sleep-related death in an inclined sleep environment

**DOI:** 10.1186/s12889-024-19731-z

**Published:** 2024-08-12

**Authors:** Laura R. Sangaré, Lance Kaufman, Robert A. Bardwell, Deborah Nichols, Mersine Bryan

**Affiliations:** 1 EpiAdvantage, Portland, OR US; 2 University of Washington, Seattle, Washington US; 3 Aegis Insight, Denver, CO US; 4 Bardwell Consulting, Denver, CO US; 5 Purdue University, West Lafayette, IN US; 6 Seattle Children’s Research Institute, Seattle, Washington US

**Keywords:** SUID, Epidemiology, Infant, Sleep, Asphyxia

## Abstract

**Background:**

Unsafe sleep environments are the primary modifiable risk factor for sudden unexpected infant death (SUID). Despite this knowledge, products that deviate from the American Academy of Pediatrics (AAP) safe sleep recommendations continue to be commonplace, such as inclined sleepers. Analyses to estimate risk among these products are lacking, perpetuating their presence in the marketplace. We present a method of comparing risk of SUID in an inclined sleeper to an AAP-recommended sleep environment.

**Methods:**

A case-control analysis using publicly available and previously published survey data was conducted for SUID events occurring between January 1, 2018 and April 12, 2019 (the date of the first inclined sleeper recall). SUID deaths were categorized as occurring in an AAP-recommended sleep environments or in an inclined sleeper. Exposure Odds Ratios (OR) are reported as the risk of SUID among infants using inclined sleepers relative to an AAP-recommended sleep environment.

**Results:**

During the study period, 4,900,573 births and 4,363 SUID deaths occurred in the US. Control characteristics were similar between previous night users of an AAP-recommended sleep environment (24%) and inclined sleepers (3.8%). Inclined sleepers were associated with a 5-fold (OR: 5.1; 95% CI: 3.2, 7.9) increased risk of SUID among infants < 12 months compared to infants in an AAP-recommended sleep environment. This risk was greatest among infants ≥ 4 months (RR: 10.4; 95% CI: 5.1, 21.5).

**Conclusions:**

This novel analysis fills a longstanding gap in risk assessments of inclined infant sleep products. More timely risk analyses may improve the safety of the marketplace.

**Supplementary Information:**

The online version contains supplementary material available at 10.1186/s12889-024-19731-z.

## Background

An estimated 3,400 sudden unexpected infant deaths (SUID) occur in the US each year [1]. An unsafe sleep environment is a nearly ubiquitous finding among infant sleep related deaths and is the strongest modifiable risk factor associated with SUID [2–8]. The American Academy of Pediatrics (AAP) has published guidelines on recommended sleep practices to reduce SUID risk including: supine sleep positioning; use of a firm, flat, non-inclined sleep surface; room sharing without bed sharing; and avoidance of soft bedding and overheating [9]. Despite these longstanding recommendations, many infant products that families use for their infant’s sleep deviate from AAP recommendations, and so may cause elevated SUID risk for users [10–14].

Each year the Consumer Product Safety Commission (CPSC) reports approximately 150 deaths among infants exposed to various nursery products, [15] such as crib bumpers, nursing pillows, inclined sleepers, and infant sleep positioners, among others. These safety signals often lay the groundwork for policy decisions including updates to the AAP guidelines on safe sleep practices and product recalls. For example, inclined sleepers such as the Rock ‘n Play sleeper was associated with over 100 infant deaths, which contributed to the development of the Safe Sleep for Babies Act of 2021 [16–18]. This act outlaws the manufacture and sale of inclined sleepers and requires all infant sleep products maintain an incline of 10 degrees or less [19]. Despite this law, numerous inclined infant products remain on the market that parents may use as infant sleep surfaces, including products such as infant swings, rockers, bouncers, loungers, among others. Establishing methods and estimates of excess risk in inclined sleeping environments are needed to consistently measure the gap between infant sleep recommendations and infant sleep practices.

Our primary aim is to present a method of assessing the risk of SUID resulting from use of an inclined sleeper. The Fisher-Price Rock ‘n Play sleeper is used to illustrate an analysis of risk of an inclined infant sleep product compared to an AAP-recommended sleep environment. The Rock ‘n Play sleeper was selected for this analysis because it was the only inclined sleeper device with data available for both exposure and fatality data.

## Methods

### Study description

We conducted a case-control analysis that included US-born infants who died unexpectedly before age 12 months between January 1, 2018, and April 12, 2019 (the date of the initial product recall) using publicly available anonymized data and previously published survey data.

### Cases: SUID events

SUID events were characterized as either (1) death in an AAP-recommended sleep environment or (2) death in a Rock ‘n Play sleeper.

1. Death in an AAP-recommended sleep environment was defined as an unexplained infant death where the infant was found supine on a firm sleep surface, and the sleep surface was free of soft objects, loose bedding, bumper pads, or any objects that could increase the risk for suffocation [6]. A firm sleep surface was defined as a crib, bassinet, portable crib or playard.
  *Data sources*: Deaths in an AAP-recommended sleep environment were estimated using a published review of SUID events in the CDC SUID Case Registry, a population-based surveillance system representing 30% of all US SUID cases that reported the proportion of unexplained SUID events among infants without unsafe sleep factors [6, 20]. An infant death was classified as SUID if infants (< 365 days old) had one of three underlying cause-of-death classifications coded per the International Classification of Diseases, 10th Revision (ICD-10): Sudden Infant Death Syndrome (SIDS, R95), unknown cause (R99), and Accidental Suffocation and Strangulation in Bed (ASSB, W75) [21–23].
  *Calculation of death counts*: Death counts were estimated by multiplying the total number of SUID deaths reported by the CDC in the study period by the estimated proportion of SUID deaths that occur in a AAP-recommended sleep environment (as reported by Parks, et al., 2021 unexplained deaths without unsafe sleep factors) (Table 1) [1, 6].

**Table 1:** Summary of estimates used in the analysis

Variable	Proportion	Count	Source(s)
Live births January 1, 2018 to April 12, 2019	100%	4,900,573	US Birth File
SUID events January 1, 2018 to April 12, 2019 Infants < 12 months	100%	4,348	US Death File
Infants 0–3 months	60%	2,622	
Infants 4–11 months	40%	1,726	
**Proportion of SUID events in an AAP recommended sleep environment**			Parks, 2021 (x) US Death File
Infants < 12 months	1.1%	48	
Infants 0–3 months	1.3%	33	
Infants 4–11 months	0.86%	15	
**Proportion of infants in an AAP recommended sleep environment in the last night with only 1 sleep device**			Bryan, 2022 (x) US Birth File
Infants < 12 months	23.7%	1,159,476	
Infants 0–3 months	30.7%	1,504,476	
Infants 4–11 months	21.9%	1,073,226	
**Count of Rock ‘n Play deaths** **January 1**,** 2018 to April 12**,** 2019**			CPSC total count (x) age and year distribution in Mannen, 2019
Infants < 12 months	0.90%	39	
Infants 0–3 months	0.40%	17	
Infants 4–11 months	0.52%	22	
**Proportion of Rock ‘n Play users in the last night with only 1 sleep device**			Bryan, 2020 (x) US Birth File
Infants < 12 months	3.8%	186,222	
Infants 0–3 months	6.8%	333,239	
Infants 4–11 months	3.1%	151,918

2) Death in a Rock ‘n Play sleeper was defined as an unexpected infant death that was associated with the Rock ‘n Play sleeper.
  *Data sources*: The number of Rock ‘n Play deaths during the study period was based on the counts of deaths associated with the Rock ‘n Play as reported by the CPSC on January 9, 2023 [17].
  *Calculation of death counts*: To estimate the number of Rock ‘n Play deaths by infant age during the study period, the total count of reported Rock ‘n Play deaths [9] was multiplied by the age and year distribution of inclined sleeper deaths occurring between 2010 and 2019 (Table 1) [17, 24].

### Controls: infants exposed to sleep environments

Control infants were selected from the underlying at-risk population that gave rise to the cases. The control population was represented by the proportion of infants exposed to either (1) an AAP recommended sleep environment using the same criteria as the SUID event or (2) a Rock-n-play sleeper.

#### Data source

A previously published nationally representative infant sleep survey of parents with infants aged ≤ 12 months conducted between March 6, 2018, and April 9, 2018 was used to establish the proportion of infants exposed to each of the sleep environments [25]. To control for varying levels of exposure time on different sleep surfaces the analysis was restricted to those respondents who reported using only one sleep surface in the previous night of sleep.

### Data analysis

Analyses were conducted using Stata version 18.0 (StataCorp., College Station, TX). Descriptive statistics were used to characterize the demographic characteristics of the survey respondents. Pearson’s Chi-squared and Fisher’s exact tests were used to assess if there were statistically significant differences in the respondents’ demographics between infants exposed only to an AAP-recommended sleep environment and those exposed only to a Rock ‘n Play. Because the risk of SUID is highest in infants < 4 months, data were analyzed for infants < 12 months, 0–3 months, and 4–11 months. The exposure odds ratio (OR) of SUID with 95% confidence intervals (CI) associated with a Rock ‘n Play sleeper compared to an AAP-recommended sleep environment is presented. Because SUID is a rare outcome, the odds ratio will approximate the relative risk in this study.

All analyses utilized deidentified public-use data sets and previously published survey data without identifiable private information and, therefore, this study was not considered as human subject research after review by the institutional review board.

Because infant sleep is not limited to nighttime, a sensitivity analysis was conducted to account for daytime use in each sleep environment. A composite variable was generated combining infants who reported using either an AAP or a Rock ‘n Play sleeper in the last night of sleep (as described above), who also reporting using only that location in the daytime.

## Results

Between January 1, 2018 and April 12, 2019, 4,900,573 births and 4,363 SUID deaths occurred in the US (Table 1) [1, 26, 27].

Control data arising from the infant sleep survey revealed most respondents reported using only one sleep surface in the previous night (75%) with the remainder of respondents reporting a maximum of two sleep surfaces. Demographic characteristics for the controls were similar between users of only an AAP-recommended sleep environment and respondents who only used a Rock ‘n Play sleeper in the previous night (Table S1).

Among controls who used only 1 sleep surface in the previous night, 24% put their infant to sleep in an AAP-recommended sleep environment, the distribution of use by infant age is presented in Table 1. Rock ‘n Play use as the only sleep surface in the previous night was reported for 3.8% of all infants, 6.8% of infants 0–3 months and 3.1% of infants 4–11 months (Table 1).

During the study period, 39 SUID cases associated with use of the Rock ‘n Play sleeper were identified along with 48 SUID cases in an AAP-recommended sleep environment (Table 2). The risk of death was 5-times greater for an infant in a Rock ‘n Play sleeper compared to an AAP-recommended sleep environment (OR: 5.1; 95% CI: 3.2, 7.9) (Table 2). The risk of a SUID event was highest among infants 4 to 11 months (OR: 10.4; 95% CI: 5.1, 21.5) and lowest for infants 0–3 months (RR: 2.3; 95% CI: 1.2, 4.3).

**Table 2:** Exposure odds ratio of SUID in a rock ‘n play sleeper compared to an AAP-recommended sleep environment by age, January 1, 2018 to April, 2019

Infants age < 12 months	SUID Cases	US Infants in General	Exposure OR (95% CI)
Rock ‘n Play Sleeper	39	186,222	5.1 (3.2, 7.9)
AAP-recommended	48	1,159,476	
**Infants ages 0–3 months**			
Rock ‘n Play Sleeper	17	333,239	2.3 (1.2, 4.3)
AAP-recommended	33	1,504,476	
**Infants ages 4–11 months**			
Rock ‘n Play Sleeper	22	151,918	10.4 (5.1, 21.5)
AAP-recommended	15	1,073,226	

OR: Odds Ratio

CI: Confidence interval

SUID: Sudden Unexpected Infant Death

AAP-recommended sleep environment is defined as an infant put to sleep in the supine position on a firm, flat, noninclined surface (i.e. crib, bassinet, or playard) without unsafe sleep items (i.e. blanket, pillow, bumper pads, stuffed animal, sleep positioner/wedge, or bottle) in the last night before the survey and used only one sleep surface

Rock ‘n Play is defined as an infant put to sleep in a Rock ‘n Play in the last night before the survey and used only one sleep surface

A sensitivity analysis accounting for daytime use in each sleep environment resulted in even stronger estimates of risk (Supplemental Table S3).

## Discussion

We conducted an epidemiological risk analysis to estimate a minimum risk of SUID in an inclined sleeper relative to an AAP-recommended sleep environment using publicly available and previously published studies. We identified that inclined sleepers were associated with at least a 5-fold increased risk of SUID compared to infants in an AAP-recommended sleep environment. This analysis confirms that inclined sleep products are an unsafe sleep environment and supports the recent policy decisions to ban the sale of inclined sleep products where the incline is steeper than 10 degrees [16]. However, numerous inclined devices remain on the market and may still be used by parents for infant sleep. Clinicians caring for infants should continue to emphasize that inclined devices are unsafe for infant sleep.

As widely reported in the literature, an unsafe sleep environment increases the risk of an asphyxiation event [2–6, 28–31]. Inclined sleep products, such as the Rock N Play, could increase the risk for sudden infant death through positional asphyxia [17, 24, 32–34]. Our analysis estimates only 24% of US infants < 12 months are sleeping in an AAP-recommended sleep environment, that includes a firm, flat, non-inclined surface, a supine position and without any unsafe sleep items. This finding is similar to previously published data from the 2016 Pregnancy Risk Assessment Monitoring System (PRAMS) and other surveys and highlights that use of unsafe sleep practices for infants continues to be common [13, 14, 35].

Although overall risk of sleep-related death is lower in infants > 4 months, we found the risk of SUID associated with use of the Rock ‘n Play to be greatest in this age group. Other publications that assessed the risk of SUID associated with use of infant sleep products were not identified. As such, the finding of an increased risk in older infants in our study cannot be compared to other studies. Based on Dr. Mannen’s biomechanical analysis, inclined sleep environments facilitate rolling which may disproportionately affect older infants resulting in a higher risk of becoming entrapped or in an asphyxial position relative to younger infants [24]. This finding highlights that inclined sleepers and other infant sleep products are still associated with elevated risk in this age group. Further studies and evaluations of safety for infant sleep related products should include all infants up to 12 months.

This analysis should be interpreted with the following limitations. First, infant sleep survey data is self-reported and, therefore, subject to social desirability bias. If respondents over-reported safe sleep practices, the resulting relative risk estimates would be spuriously high. Second, if the proportion of infant deaths in the US in an AAP-recommended sleep environment is greater than the proportion estimated from the SUID registry, the effect size of the odds ratio of SUID in an AAP approved sleep environment would be higher. However, the limited data available suggests the SUID registry is not greater than what would be expected based on previous publications [2, 3, 36–38]. Third, while the total number of unexpected infant deaths attributed to a Rock ‘n Play sleeper is unknown, we expect these deaths to be undercounted, thereby underestimating the odds ratio of SUID in Rock ‘n Play users and biasing the risk towards the null.

Despite these limitations, our study addressed several limitations of prior studies on SUID risk from infant sleep products. Deaths in AAP-recommended sleep environments were obtained from a large, multistate registry that used standardized mutually exclusive case definitions differentiating unexplained infant deaths without unsafe sleep factors from other unexplained and explained suffocation deaths [6]. Based on the detailed description provided by Parks et al., we expect there are no inclined sleeper deaths within the AAP-recommended sleep category [6]. Rather, inclined sleeper deaths could be represented in any of the other mutually exclusive categories. Recall bias and misclassification, which can arise from surveys of “usual” sleep practices, are reduced by the use of a nationally representative survey of infant sleep practices the night before completing the survey. These data allowed us to estimate an exposed population that matches the population from which the infant deaths arose. Furthermore, we were able to control for varying levels of exposure time on different sleep surfaces by restricting the analysis to those respondents who reported using only one sleep surface the previous night. We did not collect data on time of infant deaths or time of exposure to inclined sleep surfaces, and thus are not able to evaluate the impact of circadian variation on risk of death in inclined sleepers [39]. We could not identify any rationale for why deaths in an AAP-recommended sleep environment would be disproportionately under-estimated relative to deaths in inclined sleepers. Our analyses indicate that infants in an AAP-recommended sleep environment are similar to users of inclined sleepers with respect to other variables known to increase the risk of SUID including infant age, preterm delivery, birthweight, exposure to smoking, as well as parental age and race. Lastly, while the reported count of deaths in Rock ‘n Play sleepers are expected to be undercounted, this analysis still produces a valid estimate of minimum risk associated with the device.

This analytic framework may be applicable to other consumer product safety signals. However, for the approach to be valid, an accurate account of the deaths associated with the product is needed, without which the effect size may be under-estimated. This analysis was made possible by incorporating use of nationally representative survey data to estimate proportions of the population exposed to different sleep environments. Unfortunately, not all consumer products currently have available data to develop risk estimates, an ongoing limitation within CPSC data. Maintaining nationally representative product surveys across a variety of products and age groups will aid in these types of analyses becoming more mainstream. To facilitate additional studies on SUID, future surveys should collect exposure data in a way that closely matches how deaths are reported and categorized in the SUID registry. Furthermore, improved researcher access to incidence reports and transparent counts of product-related deaths are needed for more timely risk assessments. While assimilating deaths and exposures from different data sources has limitations, this analysis remains the best use of the publicly available evidence. A recent case-control analysis estimating SUID risk was reported by Parks et al., in which the authors created a pseudo-population based on the distribution of exposures identified in PRAMS and utilized the SUID registry to identify cases [40]. While that novel approach has the benefit of reporting adjusted risk estimates for available exposures of interest, the absence of data on specific infant products makes an analysis of risk not possible with that methodology for many of the problematic infant products recently, or currently, under scrutiny such as inclined sleepers, nursing pillows, and crib bumpers.

## Conclusions

We report a novel epidemiological approach using deidentified publicly available data and previously published survey data to estimate a 5-fold increased risk of SUID among infants in an inclined sleep product relative to those in an AAP-recommended sleep environment. This methodological approach may be applied to assess the risk of consumer products that deviate from AAP-sleep recommendations, as well as consumer products in general. Timely epidemiological risk analyses are needed to verify the safety of infant sleep products.

### Electronic supplementary material

Below is the link to the electronic supplementary material.

Supplementary Material 1

## Data Availability

The datasets used and/or analysed during the current study are available from the corresponding author on reasonable request.

## Abbreviations

AAP: American Academy of Pediatrics
CDC: Centers for Disease Control and Prevention
CI: Confidence Interval
CPSC: Consumer Product Safety Commission
OR: Odds Ratio
SUID: Sudden Unexpected Infant Death

## References

[1] CDC. Sudden Unexpected Infant Death and Sudden Infant Death Syndrome. Data and Statistics. https://www.cdc.gov/sids/data.htm Accessed Oct 12, 2023.

[2] Carpenter RG, Irgens LM, Blair PS, et al. Sudden unexplained infant death in 20 regions in Europe: case control study. Lancet Jan. 2004;17(9404):185–91. 10.1016/s0140-6736(03)15323-8. 10.1016/s0140-6736(03)15323-8 14738790

[3] Fleming PJ, Blair PS, Bacon C, et al. Environment of infants during sleep and risk of the sudden infant death syndrome: results of 1993-5 case-control study for confidential inquiry into stillbirths and deaths in infancy. Confidential Enquiry into Stillbirths and deaths Regional coordinators and Researchers. BMJ Jul. 1996;27(7051):191–5. 10.1136/bmj.313.7051.191. 10.1136/bmj.313.7051.191 PMC2351639 8696193

[4] Hauck FR, Herman SM, Donovan M, et al. Sleep environment and the risk of sudden infant death syndrome in an urban population: the Chicago Infant Mortality Study. Pediatr May. 2003;111(5 Pt 2):1207–14. 10.1542/peds.111.S1.1207 12728140

[5] Kemp JS, Livne M, White DK, Arfken CL. Softness and potential to cause rebreathing: differences in bedding used by infants at high and low risk for sudden infant death syndrome. J Pediatr Feb. 1998;132(2):234–9. 10.1016/s0022-3476(98)70437-8. 10.1016/s0022-3476(98)70437-8 9506633

[6] Parks SE, Erck Lambert AB, Hauck FR, Cottengim CR, Faulkner M, Shapiro-Mendoza CK. Explaining sudden unexpected infant deaths, 2011–2017. Pediatr May. 2021;147(5). 10.1542/peds.2020-035873. 10.1542/peds.2020-035873 PMC8132195 33906930

[7] Scheers NJ, Rutherford GW, Kemp JS. Where should infants sleep? A comparison of risk for suffocation of infants sleeping in cribs, adult beds, and other sleeping locations. Pediatr Oct. 2003;112(4):883–9. 10.1542/peds.112.4.883. 10.1542/peds.112.4.883 14523181

[8] Lambert AB, Shapiro-Mendoza CK, Parks SE, Cottengim C, Faulkner M, Hauck FR. Characteristics of sudden unexpected infant deaths on Shared and Nonshared Sleep surfaces. Pediatr Mar. 2024;1(3). 10.1542/peds.2023-061984. 10.1542/peds.2023-061984 PMC11117443 38374785

[9] Moon RY, Carlin RF, Hand I, Task force on sudden infant death syndrome and, the committee on fetus and newborn. Sleep-related infant deaths: updated 2022 recommendations for reducing infant deaths in the Sleep Environment. Pediatrics. 2022. 10.1542/peds.2022-057990. 35726558 10.1542/peds.2022-057990

[10] Batra EK, Teti DM, Schaefer EW, Neumann BA, Meek EA, Paul IM. Nocturnal Video Assessment of Infant Sleep environments. Pediatr Sep. 2016;138(3). 10.1542/peds.2016-1533. 10.1542/peds.2016-1533 PMC5005029 27527797

[11] Chin S, Carlin R, Mathews A, Moon R. Infant Safe Sleep practices as portrayed on Instagram: Observational Study. JMIR Pediatr Parent Nov. 2021;15(4):e27297. 10.2196/27297. 10.2196/27297 PMC8663591 34779783

[12] Gillani SH, Lowell GS, Quinlan KP. A firm recommendation: measuring the softness of infant sleep surfaces. Inj Epidemiol Sep. 2021;13(Suppl 1):30. 10.1186/s40621-021-00325-x. 10.1186/s40621-021-00325-x PMC8436463 34517913

[13] Hirai AH, Kortsmit K, Kaplan L, et al. Prevalence and factors Associated with Safe Infant Sleep practices. Pediatr Nov. 2019;144(5). 10.1542/peds.2019-1286. 10.1542/peds.2019-1286 PMC10935590 31636142

[14] Shapiro-Mendoza CK, Colson ER, Willinger M, Rybin DV, Camperlengo L, Corwin MJ. Trends in infant bedding use: National Infant Sleep position study, 1993–2010. Pediatr Jan. 2015;135(1):10–7. 10.1542/peds.2014-1793. 10.1542/peds.2014-1793 PMC4279068 25452654

[15] Yang T, United States Consumer Product Safety Commission. Injuries and deaths associated with nursery products among children younger than age five. August, 2023. https://www.michigan.gov/lara/-/media/Project/Websites/lara/CCLB/Providers/Nursery-Products-Annual-Report-2023.pdf?rev=518471c63ab04467b2792a5a7b62b2e3&hash=934B4766FBE00E133028B133EA8D8337 Accessed Oct 14, 2023.

[16] The White House, Bill HR. 3182, the Safe Sleep for Babies Act of 2021. https://www.whitehouse.gov/briefing-room/legislation/2022/05/16/bills-signed-h-r-3182-and-h-r-6023/ Accessed on November 23, 2023.

[17] CPSC. US Consumer Product Safety Commision. Fisher-Price Reannounces Recall of 4.7 Million Rock ‘n Play Sleepers; At Least Eight Deaths Occurred After Recall. https://www.cpsc.gov/Recalls/2023/Fisher-Price-Reannounces-Recall-of-4-7-Million-Rock-n-Play-Sleepers-At-Least-Eight-Deaths-Occurred-After-Recall Accessed Oct 9, 2023.

[18] CPSC. US Consumer Product Safety Commision. Fisher-Price Recalls Rock ‘n Play Sleepers Due to Reports of Deaths. https://www.cpsc.gov/Recalls/2019/Fisher-Price-Recalls-Rock-n-Play-Sleepers-Due-to-Reports-of-Deaths Accessed Oct 11, 2023.

[19] CPSC. Consumer Product Safety Commision. 16 CFR Part 1310 [CPSC Docket No. 2022–0025]. Federal Register. Vol. 88, No. 157, Wednesday. August 16, 2023. Ban of Inclined Sleepers for Infants. https://www.govinfo.gov/content/pkg/FR-2023-08-16/pdf/2023-17350.pdf Accessed March 7, 2024.

[20] CDC. SUID and SDY Case Registry. https://www.cdc.gov/sids/case-registry.htm Accessed 10/28/23.

[21] National Center for Health Statistics. National Vital Statistics System. Instructions for classifying the underlying cause of death. NCHS Instruction Manual; part 2a. Hyattsville, MD. https://www.cdc.gov/nchs/nvss/instruction_manuals.htm Accessed Oct 9, 2023.

[22] National Center for Health Statistics. National Vital Statistics System. Instructions for classifying the underlying cause of death. NCHS Instruction Manual; part 2b. Hyattsville, MD. https://www.cdc.gov/nchs/nvss/instruction_manuals.htm Accessed Oct 9, 2023.

[23] World Health Organization. International Statistical Classification of Diseases and related health problems. Geneva, Switzerland: World Health Organization; 1992.

[24] Mannen EM, Carroll JL, Bumpass DB et al. Biomechanical Analysis of Inclined Sleep Products – FINAL Report 09.18.2019, Redacted. https://www.cpsc.gov/s3fs-public/Dr-Mannen-Study-FINAL-Report-09-18-2019_Redacted.corrected_0.pdf?g.Jao0IN_zU.TjiX4FeSUM3SPc3Zt_25 Accessed Oct 9, 2023.

[25] Bryan MA, Florence A, Gower AD, Evans YN, Moreno MA. Safe sleep behaviors and factors Associated with Infant Second Sleep practices. Pediatr May. 2022;30. 10.1542/peds.2021-053935. 10.1542/peds.2021-053935 35634879

[26] Martin JA, Hamilton BE, Osterman MJK, Driscoll AK. Births: final data for 2018. Natl Vital Stat Rep Nov. 2019;68(13):1–47. 32501202

[27] Martin JA, Hamilton BE, Osterman MJK, Driscoll AK. Births: final data for 2019. Natl Vital Stat Rep Apr. 2021;70(2):1–51. 33814033

[28] Patel AL, Harris K, Thach BT, Inspired. CO(2) and O(2) in sleeping infants rebreathing from bedding: relevance for sudden infant death syndrome. *J Appl Physiol (*1985*)*. Dec 2001;91(6):2537-45. 10.1152/jappl.2001.91.6.2537 10.1152/jappl.2001.91.6.2537 11717216

[29] Tappin D, Ecob R, Brooke H. Bedsharing, roomsharing, and sudden infant death syndrome in Scotland: a case-control study. J Pediatr Jul. 2005;147(1):32–7. 10.1016/j.jpeds.2005.01.035. 10.1016/j.jpeds.2005.01.035 16027691

[30] Wong FY, Witcombe NB, Yiallourou SR, et al. Cerebral oxygenation is depressed during sleep in healthy term infants when they sleep prone. Pediatr Mar. 2011;127(3):e558–65. 10.1542/peds.2010-2724. 10.1542/peds.2010-2724 21357341

[31] Carlin RF, Moon RY, Risk, Factors. Protective factors, and current recommendations to reduce Sudden Infant Death Syndrome: a review. JAMA Pediatr Feb. 2017;1(2):175–80. 10.1001/jamapediatrics.2016.3345. 10.1001/jamapediatrics.2016.3345 27918760

[32] Siddicky SF, Bumpass DB, Krishnan A, Tackett SA, McCarthy RE, Mannen EM. Positioning and baby devices impact infant spinal muscle activity. J Biomech May. 2020;7:104:109741. 10.1016/j.jbiomech.2020.109741. 10.1016/j.jbiomech.2020.109741 PMC7188598 32178849

[33] Wang J, Siddicky SF, Carroll JL, et al. Do inclined sleeping surfaces impact infants’ muscle activity and movement? A safe sleep product design perspective. J Biomech Oct. 2020;9:111:109999. 10.1016/j.jbiomech.2020.109999. 10.1016/j.jbiomech.2020.109999 32862027

[34] Wang J, Siddicky SF, Carroll JL, et al. Infant inclined sleep product safety: a model for using biomechanics to explore safe infant product design. J Biomech Nov. 2021;9:128:110706. 10.1016/j.jbiomech.2021.110706. 10.1016/j.jbiomech.2021.110706 34624615

[35] Melia KL, Jenkins JL. Durable Nursery Products Exposure Survey (DNPES). Final Summary Report. November 2014. https://www.cpsc.gov/s3fs-public/DurableNurseryProductsExposureSurveyDNPESFinalSummaryReport2014.pdf?VersionId=5R29kvpEAB20KnHt6tFzyTXsKxTvRJsM Accessed Oct 12, 2023.

[36] Kappy B, Edmunds K, Frey M, et al. Emergency Department visits before sudden unexpected infant death: a touchpoint for unsafe sleep reduction. Acad Pediatr Aug. 2022;22(6):1065–72. 10.1016/j.acap.2022.03.009. 10.1016/j.acap.2022.03.009 35307602

[37] Brooke H, Gibson A, Tappin D, Brown H. Case-control study of sudden infant death syndrome in Scotland, 1992-5. *BMJ*. May 24. 1997;314(7093):1516-20. 10.1136/bmj.314.7093.1516 10.1136/bmj.314.7093.1516 PMC2126747 9169398

[38] Taylor JA, Krieger JW, Reay DT, Davis RL, Harruff R, Cheney LK. Prone sleep position and the sudden infant death syndrome in King County, Washington: a case-control study. J Pediatr May. 1996;128(5 Pt 1):626–30. 10.1016/s0022-3476(96)80126-0. 10.1016/s0022-3476(96)80126-0 8627433

[39] Anderson TM, Allen K, Ramirez JM, Mitchell EA. Circadian variation in sudden unexpected infant death in the United States. Acta Paediatr May. 2021;110(5):1498–504. 10.1111/apa.15695. 10.1111/apa.15695 PMC8246563 33251652

[40] Parks SE, DeSisto CL, Kortsmit K, Bombard JM, Shapiro-Mendoza CK. Risk factors for suffocation and unexplained causes of infant deaths. Pediatr Jan. 2023;1(1). 10.1542/peds.2022-057771. 10.1542/peds.2022-057771 PMC9942004 36464994

