# Supplementary material for: The risk of sleep-related death in an inclined sleep environment
Source: BMC Public Health. 2024 Aug 12;24:2186. doi: 10.1186/s12889-024-19731-z (PMC11320768; doi:10.1186/s12889-024-19731-z)
Supplement: Supplementary file 1 — Supplementary Material 1 [file 12889_2024_19731_MOESM1_ESM.docx]

Supplemental Table S1: Demographic characteristics from the infant sleep survey, all respondents and users reporting only 1 sleep surface in the last night in an AAP recommended sleep environment or a Rock ‘n Play sleeper

|  | **Total Population**  **n=1,500** | **AAP-recommended**  **n=248** | | **Rock ‘n Play**  **n=40** | **p-value*** |
| --- | --- | --- | --- | --- | --- |
| **Infant characteristics** | | |  |  |  |
| ***Infant age at time of survey (months)*** |  |  | |  |  |
| 0-3 | 23.9% | 25.4% | | 35% | 0.2 |
| 4-6 | 30.6% | 31.9% | | 43% | 0.2 |
| 7-12 | 45.5% | 42.7% | | 23% | 0.02 |
| ***Infant gender, Male*** | 54.0% | 49.6% | | 48% | 0.8 |
| ***Birthweight (lbs)*** |  |  | |  |  |
| <6 | 21.4% | 19.4% | | 23% | 0.2 |
| 6-7 | 27.2% | 27.8% | | 18% |  |
| 7-8 | 28.4% | 28.2% | | 23% |  |
| ≥9 | 22.7% | 24.6% | | 38% |  |
| ***Preterm birth*** | 16.1% | 11.7% | | 8% | 0.4 |
| **Parent characteristics** | | |  |  |  |
| ***Female respondent*** | 74.2% | 82.2% | | 83% | 0.6 |
| ***Parental age (years)*** |  |  | |  |  |
| ≤24 | 16.4% | 7.7% | | 10% | 0.1 |
| 25-34 | 60.2% | 72.2% | | 65% |  |
| 35+ | 23.4% | 20.2% | | 25% |  |
| ***Parental race*** |  |  | |  |  |
| White Non-Hispanic | 64.5% | 74.6% | | 83% | 0.3 |
| Black Non-Hispanic | 12.4% | 8.5% | | 3% |  |
| Hispanic | 17.3% | 12.1% | | 15 |  |
| Other | 5.7% | 4.8% | | 0% |  |
| ***Parental education*** |  |  | |  |  |
| High school or less | 21.4% | 12.5% | | 23% | 0.1 |
| Some collage | 32.5% | 27.0% | | 38% |  |
| 4-yr degree | 26.3% | 33.1% | | 28% |  |
| Graduate school or more | 19.3% | 27.4% | | 13% |  |
| ***Currently married*** | 77.2% | 83.5% | | 90% | 0.3 |
| ***Current smoker in home, %*** | 18.2% | 8.9% | | 15% | 0.2 |
| ***First child*** | 39.2% | 43.6% | | 25% | 0.03 |
| ***Region*** |  |  | |  |  |
| Midwest | 20.8% | 25.8% | | 13% | 0.2 |
| Northeast | 21.8% | 21.0% | | 25% |  |
| South | 32.1% | 29.8% | | 43% |  |
| West | 23.1% | 23.4% | | 20% |  |

Variable proportions do not add to 100 due to rounding

*p-value based on chi-squared tests between safe sleepers and Rock ‘n Play users.

AAP-recommended sleep environment is defined as an infant put to sleep in the supine position on a firm, flat, noninclined surface (i.e. crib, bassinet, or playard) without unsafe sleep items (i.e. blanket, pillow, bumper pads, stuffed animal, sleep positioner/wedge, or bottle) in the last night before the survey and used only one sleep surface.

Rock ‘n Play is defined as an infant put to sleep in a Rock ‘n Play in the last night before the survey and used only one sleep surface.

Supplemental Table S2: Comparison of variable definitions by data source

| **Analytic variable** | **Infant Sleep Survey**  **Bryan, 2022** | **SUID Registry**  **Parks, 2021** |
| --- | --- | --- |
| Study period  January 1, 2018 to April 12, 2019 | March 6, 2018, and April 9, 2018. | 2011 to 2017 |
| Infant Age | Infant age at time of survey | Infant age at death |
| Infant gender | What is your baby’s gender? | Infant gender (death certificate) |
| Sleep position | Now think about LAST NIGHT. What position did you place your baby to sleep in last night?  Response options (side, stomach, back, other) | Infant placed to sleep at incident: back versus  stomach, side |
| Unsafe sleep items | Now think about LAST NIGHT. What items did your baby have in their sleep space last night?  Response options (blanket, pillow, pacifier, bumper pads, stuffed animal, sleeper positioner/wedge, wearable baby monitor, bottle, other) | The following objects were not in the infant’s sleeping area: comforter, quilt, thin blanket or flat sheet, pillow, cushion, U-shaped pillow, sleep positioner, bumper pads, toys versus at least 1 of the above objects was present in the infant’s sleeping area at the incident |
| Sleep surface | Now think about LAST NIGHT. Where did your baby sleep last night?  Response options (crib, bassinet, cradle, playard, Rock-N-Play, swing, adult bed or mattress, sofa, carseat, co-sleeper, other) | Incident sleep place was a crib or bassinet versus an adult bed, waterbed, playpen, or other play structure but not portable crib, chair, floor, car seat, stroller, or futon |

Table S3: Sensitivity Analysis. Exposure odds ratio of SUID in a Rock ‘n Play sleeper compared to an AAP-recommended sleep environment by age, restricted to last night and daytime users

| **Infants age < 12 months** | **SUID Cases** | **US Infants in General** | **Exposure OR (95% CI)** |
| --- | --- | --- | --- |
| Rock ‘n Play Sleeper | 39 | 42,145 | 7.6 (4.9, 11.5) |
| AAP-recommended | 48 | 391,066 |  |
| **Infants ages 0-3 months** | | | |
| Rock ‘n Play Sleeper | 17 | 29,893 | 6.4 (3.6, 11.6) |
| AAP-recommended | 33 | 373,424 |  |
| **Infants ages 4-11 months** | | | |
| Rock ‘n Play Sleeper | 22 | 45,575 | 12.8 (6.6, 24.6) |
| AAP-recommended | 15 | 396,456 |  |

AAP-recommended sleep environment is defined as an infant put to sleep in the supine position on a firm, flat, noninclined surface (i.e. crib, bassinet, or playard) without unsafe sleep items (i.e. blanket, pillow, bumper pads, stuffed animal, sleep positioner/wedge, or bottle) in the last night before the survey and used only one sleep surface and also used only a crib, bassinet, or playard in the daytime.

Rock ‘n Play is defined as an infant put to sleep in a Rock ‘n Play in the last night before the survey and used only one sleep surface and also used only a Rock ‘n Play sleeper in the daytime.
